# Ecological predictors of daily dietary intake in black adolescents with overweight and obesity in the families improving together (FIT) for weight loss trial

**DOI:** 10.1007/s10865-026-00686-y

**Published:** 2026-07-01

**Authors:** Taylor R. White, Dawn K. Wilson, Sonali Tucker, Allison Sweeney

**Affiliations:** 1 Department of Psychology, University of South Carolina, Columbia, SC USA; 2 School of Nursing, University of South Carolina, Columbia, SC USA

**Keywords:** Black or African American, Adolescent, Diet, Ecological, Food availability, Obesity

## Abstract

Healthful eating habits (i.e., increased fruit and vegetable and decreased hyperpalatable energy-dense food and drink consumption) are important for preventing chronic disease risk (e.g., obesity) and improving overall health in Black adolescents.This study examines the intrapersonal, interpersonal, and environmental factors associated with daily dietary intake in Black adolescents with overweight and obesity. Baseline data were analyzed from 241 Black parent-adolescent dyads who participated in the Families Improving Together (FIT) for Weight Loss trial (Adolescent: *M*age = 12.8 years, *SD* = 1.8; 63% female; *M*BMI% = 96.6, *SD* = 4.3). Intrapersonal factors included dietary motivation. Interpersonal factors included social support for diet from family. Environmental factors included access to food in the home. Dietary outcomes were measured using validated adolescent self-reported surveys. Separate multiple linear regressions revealed several associations between ecological factors and daily food intake. Greater access to more-healthful foods was associated with increased daily vegetable intake (*β* = 0.18, *SE* = 0.08, *p *< .05), whereas greater access to less-healthful foods was associated with increased daily fruit intake (*β* = 0.24, *SE* = 0.08, *p *< .05). Greater access to less-healthful foods was associated with increased daily energy-dense food intake (*β* = 0.75, *SE* = 0.10, *p *< .001), whereas increased self-reported dietary motivation was associated with decreased daily energy-dense food intake (*β *= -0.24, *SE* = 0.10, *p *< .05). Greater access to less-healthful foods (*β* = 1.48, *SE* = 0.23, *p *< .001) and higher adolescent BMI (*β* = 0.84, *SE* = 0.40, *p *< .05) was also associated with increased sugar-sweetened beverage consumption. These findings suggest that access to more-healthful foods is associated with greater vegetable intake, while restricting access to less-healthful foods and increased motivation for more healthful eating were associated with lower sugar-sweetened beverage and energy-dense food consumption among Black adolescents. These findings may inform future health promotion programs across various ecological levels of dietary behaviors in Black youth with overweight and obesity.

## Introduction

Black or African Americans adolescents are vulnerable for risk factors of poor nutrition (Xu et al., 2019). Poor nutrition increases the risk for chronic diseases, such as obesity, type 2 diabetes, heart disease, as well as mental health disorders, such as depression (Blasco et al., 2020; Gong & Lai, 2025; Wang et al., 2023). Healthful eating habits, such as eating more fruit and vegetables and consuming less hyperpalatable energy-dense foods (high fat, high sugar) and sugar-sweetened beverages contribute to preventing chronic disease risk (e.g., obesity, diabetes) and improving health in Black adolescents (Ducharme-Smith et al., 2021). Approximately forty-percent of Black adolescents have overweight or obesity (Ogden et al., 2014), and these youth are at an increased risk for obesity-related risks due to unhealthful dietary outcomes, including cardiovascular and metabolic diseases (Fitzpatrick et al., 2013; Vyas et al., 2023). Thus, the purpose of this study is to examine the ecological determinants of daily dietary intake patterns in Black adolescents with overweight and obesity.

Social-ecological theory posits that dietary intake in Black adolescents is influenced by ecological factors at the interpersonal, intrapersonal, and environmental levels (Bronfenbrenner, 1992; Wilson et al., 2017). These factors include motivation for more healthful eating (intrapersonal), receiving social support from family for more healthful eating (interpersonal), and having greater access and availability to more-healthful and less access and availability to less-healthful foods in the home (environmental) (Wilson et al., 2017). Findings from systematic reviews of qualitative studies have identified these factors as predictors of diet quality in adolescents from sociodemographic minority groups, including underserved African American households (Bel-Serrat et al., 2022; Eicher-Miller et al., 2023; Mekonnen et al., 2020). Despite the well-documented literature on ecological influences of dietary intake in adolescents, no study has assessed the combined influence of these specific predictors of daily dietary outcomes of fruit and vegetable intake and hyperpalatable energy-dense foods in a sample exclusively of Black adolescents with overweight and obesity.

Findings from our research group’s Families Improving Together (FIT) family-based, weight-loss trial for Black adolescents with overweight and obesity demonstrate that dietary motivation, social-support, and access to foods in the home are positively associated with more frequent and greater quality of family mealtime over time (Loncar et al., 2024; Quattlebaum et al., 2025; Wilson et al., 2021); however, these outcomes have not included adolescent self-reported daily dietary outcomes of fruit and vegetable intake or energy dense foods and sweetened beverages. Wilson and colleagues (2021) demonstrated that higher levels of parental responsiveness and lower levels of parental demandingness were associated with increased frequency of weekly family mealtimes over 16 weeks in families participating in the group-based motivational plus family weight loss intervention (Wilson et al., 2021). Similarly, Loncar and colleagues (2024) demonstrated that higher levels of parental responsiveness and parental responsibility were associated with more frequent family meals, and higher levels of parental responsiveness and lower levels of parental demandingness predicted higher quality family mealtimes from baseline to 16 weeks (Loncar et al., 2024). Quattlebaum and colleagues (2025) examined family mealtime outcomes, but not specifically dietary intake of fruit and vegetable intake, energy dense foods, or sweetened beverages, across multiple ecological levels, including adolescent dietary motivation, perceived social support from family for diet, and perceived access and availability to more-healthful foods in the home (Quattlebaum et al., 2025). Findings revealed that higher levels of social support from family for diet was associated with higher quality of family mealtimes over 16-weeks (Quattlebaum et al., 2025). Taken together, these studies demonstrate that multiple ecological level influences at the intrapersonal, interpersonal, and environmental level influence on family mealtime outcomes in Black adolescents with overweight and obesity, but little past attention on the specific dietary outcomes that these adolescents may be consuming. This study will extend current literature by evaluating the relationship between dietary motivation, social support for diet, and access to more-healthful and less-healthful foods specifically related to fruits and vegetables and high fat and high sugar foods and drinks in the home in a sample of exclusively Black adolescents with overweight and obesity.

Autonomous dietary motivation has been linked to healthier dietary intake in adolescents (Wilson et al., 2017). Consistent with Ryan and Deci’s (2000) theory on motivation, autonomous motivation for diet involves agency in making healthful dietary decisions that are consistent with personal values and goals, competency in engaging in healthful eating behaviors, such as acquiring the knowledge and skills to eat healthful foods and feeling socially connected to others while engaging in healthful dietary behaviors (Ryan & Deci, 2000). Studies have demonstrated that higher levels of autonomous motivation have been association with higher levels of fruit and vegetable intake and lower levels of energy dense foods and sugar-sweetened beverages over a seven-day period in adolescents (Parks et al., 2018; Zhang et al., 2020).

Social support from friends and family is another important ecological predictor of dietary intake in adolescents. Family Systems Theory (FST; Broderick, 1993) postulates that parenting that encourages adolescent autonomy around diet, including responsive parenting styles and authoritative parenting practices, are associated with healthier adolescent dietary behaviors (Larson et al., 2015; Wilson et al., 2017; Wrobleski et al., 2018). Parents also dictate the availability of foods in the home, set routines and expectations around eating, including regular and consistent mealtimes, and focal in shaping the food environment of the home and dictating the access and availability of more-healthful and less-healthful foods (Murillo et al., 2024). While social support has been an important predictor of diet, few studies have examined this factor in Black adolescents with overweight and obesity while considering other ecological factors related to daily dietary outcomes.

Environmental factors also shape dietary intake, including the availability of foods in the home. Difficulties accessing and limited availability of healthful foods in the home may limit adolescents’ opportunities to engage in healthier eating. Black families are exposed to several structural and built environmental factors that negatively impact access to healthful foods, including income inequality and food insecurity (Kraft et al., 2020; Samuel et al., 2023; Walker et al., 2021). Black families are more likely to eat pre-prepared foods and fast food at meals compared to homemade foods (Berge et al. 2016a). Black families are more likely to be enrolled in food assistance programs (e.g., SNAP, WIC; Samuel et al., 2023) and studies have demonstrated that children in families receiving public assistance for food are more likely to consume fruits and vegetables (Futrell Dunaway et al., 2017), while some studies suggest that some families receiving governmental assistance are less likely to consume fruits and vegetables (Engel & Ruder, 2020). In a study of Black adolescents living in urban areas, more foods purchased at fast food locations by the household predicted less intake of vegetables (Trude et al., 2016). Other contextual factors include access to grocery stores or other places to purchase fresh fruits and vegetables (McCullough et al., 2022; Sheats et al., 2014), which in turn may predict the availability of fruits and vegetables available in the home. Access and availability of less-healthful and more-healthful foods in the home are important for understanding the other influences of dietary motivation and social support on adolescent dietary outcomes.

Previous research has examined ecological predictors of adolescent-reported daily dietary intake across several diet outcomes (fruits and vegetables, energy dense foods, sugar-sweetened beverages); however, few studies have examined the multiple influence of dietary motivation, social support for diet from family, and access and availability of food in the home on specific dietary measures or assessed for these relationships in primarily Black adolescents with overweight and obesity. Di Noia and Byrd-Bredbenner (2013) demonstrated that higher consumption of fruits and vegetables was predicted by higher levels of family support and availability of fruits and vegetables in the home, while also considering other factors in the model, such as taste preferences, Afrocentric values, and interactions with family support and availability in a sample of African American adolescents and their parents (*n* = 93) (Di Noia & Byrd-Bredbenner, 2013). Another study by Neurmark-Stainzer and colleagues (2003) examined several ecological factors, including autonomous dietary motivation factors, social support from parents, and access and availability of foods in the home, however and despite the large sample size (*n* = 4,746 adolescents), only 19.0% of the sample was African American (Neumark-Sztainer et al., 2003). Other studies have also not focused solely on Black adolescents with overweight and obesity. For example, a study by Loth and colleagues (2016) demonstrated that the availability of more healthful foods in the home predicted increased consumption of fruit and vegetable intake, whereas higher levels of parent modeling predicted lower sugar-sweetened beverage intake and snack foods among adolescents when considering the combined effects of parent support and availability of foods in the home in predominantly white adolescents (*n* = 2,383, 23.1% African American) (Loth et al., 2016). One study demonstrated that parent encouragement and modeling and accessibility and availability of fruit and vegetables in the home were positively associated with adolescents having more daily servings of fruit and vegetables regardless of if a family meal occurred that day, again in predominately white adolescents (*n* = 2,491, 35.6% African American) (Watts et al., 2017). Another study found that several parenting support for diet, including autonomy support, monitoring, and expectations, as well as availability of foods in the home, predicted dietary outcomes among adolescents, however, adolescent motivation for diet was not examined again in predominantly white adolescents (*n* = 622, 27.5%) (Reicks et al., 2023). This current study examines autonomous motivation for diet (intrapersonal), social support for diet (interpersonal), and access and availability of foods in the home (environmental) and intake of daily fruits and vegetables, energy dense foods (high fat, high sugar), and sugar-sweetened beverage consumption in Black adolescents with overweight and obesity.

The purpose of this study, thus, is to examine the ecological determinants of daily dietary intake patterns that focus on specific dietary factors of fruit and vegetable intake, as well as energy dense foods and sweetened beverages in Black adolescents with overweight and obesity. Informed by the social-ecological model, Family Systems Theory, and motivation theory, it is hypothesized that higher levels of dietary motivation are associated with more self-reported daily intake of fruit and vegetables and lower self-reported daily consumption of energy dense foods and sugar-sweetened beverages. It is also hypothesized that higher levels of social support for healthful eating, greater access to more-healthful food, and lower access to less-healthful foods in the home are associated with more self-reported daily intake of fruits and vegetables and lower self-reported daily consumption of energy dense foods and sugar-sweetened beverages.

## Methods

### Study sample

This study analyzed secondary data from the FIT trial (Wilson et al., 2015, 2022). Baseline data was collected from 241 African American adolescent-parent pairings and were used for analyses in the present study. More detailed information on the design of the FIT trial (Wilson et al., 2015) and the primary (Wilson et al., 2022) and secondary outcomes (Wilson et al., 2021) have been reported elsewhere.

The purpose of the FIT trial (Wilson et al., 2015, 2022) was to evaluate the efficacy of a motivational plus family-based weight-loss (M + FWL) intervention versus a comprehensive health education program on reducing body mass index (BMI) and improving diet and physical activity in African American adolescents with overweight and obesity and their parent (ClinicalTrials.gov ID#: NCT01796067). Phase 1 of the trial tested the efficacy of an 8-week face-to-face group-based M + FWL intervention program (versus a comprehensive health education program). In phase 2 of the trial, participants were re-randomized to either an 8-week tailored online intervention or a control online program resulting in a 2 (M + FWL vs. comprehensive health education group) x 2 (intervention vs. control). The FIT trial was approved by the University of South Carolina Institutional Review Board.

FIT participants were recruited between 2013 and 2018 and follow-up measures for the final cohort concluded in Spring of 2019. Adolescents were eligible for participation if they (1) identified as African American, (2) met criteria for overweight or obesity (BMI ≥ 85th percentile), (3) were between 11 and 16 years old, (4) had internet access, and (5) had a parent willing to participate in the study. The FIT inclusion criteria were extended to include participants between the 70th and 85th BMI percentile for these secondary data analyses. Six participants were between the 70th and 85th percentile for BMI and were included in the analyses (4 of which were 80th percentile or above), as this BMI range is associated with increased risk for adolescents transitioning to overweight and obesity (Kimm et al., 2002; Ng & Cunningham, 2020). Exclusion criteria included having a medical or psychiatric condition that may interfere with physical activity or diet, taking medications that could impact weight or appetite, or current enrollment in another structured weight-loss program. Participants were recruited through local clinics, schools, and community centers in Columbia, SC (i.e., churches and recreational centers) using culturally relevant recruitment approaches (Huffman et al., 2016). Culturally relevant recruitment approaches included culturally-relevant ads (African American publications), community partners that serve African American families (Boys and Girls Club), and sociocultural events such as Black History Month community events and events at African American churches. Prior to participation in the study, adolescents and their parents provided written informed assent and consent, respectively.

### Measures

**Demographics and covariates**. Demographic information was collected through self-report surveys through the adolescent and parent at the baseline visit before the start of the trial. These surveys included age, sex, and parent yearly income. Age was calculated at the time of baseline measurement using the birth date of the child and the date of the measurement appointment. Parents reported annual household income by selecting one of the following categories: Less than $10,000, $10,000 to $24,999, $25,000 to $39,999, $40,000-$54,999, $55,000 to $69,999, $70,000 to $84,999, or $85,000 or more. Parent marital status was measured using the following categories: Married, Separated, Divorced, Widowed, Never Married, or In an unmarried couple.

**Baseline anthropometrics**. Height and weight were measured by trained research staff for both the adolescent and the parent and taken at the baseline visit. Two measures of height and weight were gathered using a Shorr height board and SECA 880 digital scale. A third measure was taken if the first two measurements differed by 1.0 cm or 0.5 kg. An average of these measures was calculated, and zBMI was assessed using the Center for Disease Control (CDC) standard for adolescents.

### Primary outcome measure

**Daily dietary intake**. Daily servings of fruits and vegetables, hyperpalatable energy-dense (high fat, high sugar) foods, and sugar-sweetened beverages were assessed through self-reported questionnaires (Prochaska & Sallis, 2004). Dietary intake was assessed with a single item for each food type (fruits and vegetables, energy-dense foods, and sugar-sweetened beverages) measuring the typical number of daily servings of these foods. Fruit and vegetable intake was assessed using a validated measure which has previously demonstrated moderate test-retest reliability (ICC = 0.80) and validity with three-day food records (Spearman’s *r* = .23, *p* = .008) (Prochaska & Sallis, 2004). Participants were given examples of fruits and vegetables, for example, 1 medium piece of fresh fruit or ½ cup of fruit salad was considered a single serving of fruit, and 1 medium carrot or other fresh vegetable was considered a single serving of vegetables. Response choices ranged from “none” to “four or more” for daily servings of fruits and vegetables. Daily energy-dense food intake was adapted from the validated fruit and vegetable questionnaire. Adolescents were asked to rate their daily “junk food” consumption, which included foods such as candies and chips, on a response scale from “none” to “five or more” servings. Participants also reported on their daily servings of sugar-sweetened beverages by reporting on their intake of sweet tea, soda, and fruit drinks for each beverage separately on a response scale from “none” to “five or more” servings. Total daily intake of sugar-sweetened beverages was calculated as a summation of sweet tea, soda, and fruit drink intake.

### Ecological predictors

**Dietary motivation**. Dietary motivation was measured with 8 items that assessed the adolescent’s intrinsic motivation for more healthful eating. This scale was developed by Wilson and colleagues (2002) and then adapted to improve psychometric properties (Lawman et al., 2011, 2012; St George et al., 2013). Responses were rated on a 6-point scale from to diet to be rated on a scale ranging “strongly disagree” to “strongly agree” (Wilson et al., 2002). Sample items included “Eating healthy is important to me” and “I am excited about eating healthy on most days.” This scale demonstrated high internal reliability in the current study, α = 0.89, and Cronbach’s alphas in previous studies have ranged from 0.82 to 0.88 (Lawman et al., 2011, 2012; St George et al., 2013).

**Social support for diet from family**. Social Support for Diet from Friends was measured with 12 items that assessed the adolescent’s perceived support for diet from family. Survey items were adapted from the Support for Diet and Exercise Behaviors Scale (Sallis et al., 1987). Negatively worded items were removed, and positive worded items were used to assess adolescent perceptions of support for diet from family. Responses were rated on a 5-point scale from “none” to “very often” (Sallis et al., 1987). Sample items included “In the past month, my family (or members of my household) complimented me on my eating habits (“Keep it up”, “We are proud of you”). This scale demonstrated high internal reliability in the current study, α = .85. This scale also demonstrated construct validity in prior studies with youth (Kitzman-Ulrich et al. 2010; Sallis et al. 1988a, b, 1999).

**Food access and availability in the home**. Food Access and Availability in the Home was assessed with a 17-item self-report scale (Ding et al. 2012). This scale assessed how often specific types of food are available in the adolescent’s home. Two subscales examined the availability of less-healthful foods and more-healthful foods (Ding et al. 2012). Less-healthful foods included cakes, candies, and sodas. More-healthful foods included raw vegetables and raw fruit. Participants reported on a 5-point Likert-scale from “never” to “always.” Higher scores represented greater access and availability of foods in the home. In the current study, both subscales achieved high internal reliability as measured by Cronbach’s alpha (less-healthful foods: α = 0.77, more-healthful foods: α = 0.69). Previous studies also reported moderate to high internal consistency in both subscales (less-healthful foods: α = 0.62, more-healthful foods: α = 0.75) (Ding et al. 2012).

### Data analysis

Baseline data taken prior to beginning the intervention was used for analyses. Four separate multiple linear regressions examined associations between ecological factors and daily dietary intake with adolescent age, adolescent sex, adolescent zBMI, parent marital status, and parent yearly income included as covariates in each model. These covariates were selected due to known associations with dietary outcomes (Loncar et al., 2024; Quattlebaum et al., 2021; Wilson et al., 2021). Ecological predictor variables were selected consistent with the ecological framework that guided the proposed study. Predictors variables, including ecological predictors and covariates, were concurrently entered into each model. Ecological predictor variables were normed at the item level using z-score transformations before being averaged to ensure that each item contributed equally to the overall composite score. Adolescent age at the time of data collection was coded in years and mean centered. Sex was coded ‘1’ for males and ‘0’ for females. Parent marital status was coded “1” for married and “0” for all other responses. Consistent with our previous studies, parent yearly income was coded “1” for income $40,000 or greater and “0” for income less than or equal to $39,999. Bivariate correlations were performed to examine associations between study variables.

Missing data was assumed to be missing at random. Multiple imputations were used to address missing data for all covariate, predictor, and outcome variables by using the MICE package in R statistical software package, version 4.2.2 (The R Foundation for Statistical Computing, Vienna, Austria).

All statistical analyses, including assumptions for the multiple regression analyses and case diagnostics were assessed using R. To address the assumption of normality, histograms of the standardized residuals were assessed. Scatterplots of the standardized residuals and predicted values were evaluated to assess for homoscedasticity. A Durbin-Watson test was used to assess for independence of errors. A Cook’s distance measure was used to check for influential points in the data. Bivariate correlations were used to examine assumptions of multicollinearity among independent variables.

## Results

### Demographics

Baseline data was collected from 241 African American parent-adolescent dyads participating in the FIT trial (Wilson et al., 2022). Table 1 outlines the descriptive data from the current sample and the larger FIT sample. The current study sample included 241 African American parent-adolescent dyads. Adolescent and parent participants were primarily female (adolescent: 64%; parent: 96%). The mean age for adolescents was 12.8 (*SD* = 1.8) and the mean BMI percentile was 96.6 (*SD* = 4.3, Range = 70.5–99.9). The majority of families reported annual household income as below $40,000 (61.8%) and 65.6% of parents were not married.

**Table 1:** Demographic characteristics and study variables, *n* = 241

Variable	Value
Adolescent age (years) *M (SD)*	12.83 (1.75)
Adolescent sex (female), *N%*	153 (64%)
Adolescent BMI percentile *M (SD*), Range	96.61 (4.25), 70.5–99.9
Parent age *M (SD)*	43.18 (8.65)
Parent education, *N%*	
9 to 11 years	6 (2.5%)
12 years	32 (13.3%)
Some college	99 (41.1%)
4 year college	45 (18.7%)
Professional	55 (22.8%)
Parent income, *N%*	
Less than $10,000	36 (14.9%)
$10,000-$24,000	49 (20.3%)
$25,000-$39,000	64 (26.6%)
$40,000-$54,000	31 (12.9%)
$55,000-$69,000	21 (8.7%)
$70,000-$84,000	12 (5.0%)
$85,000 or greater	24 (10.0%)
Parents married, *N (%)*	83 (34.4%)
Parent BMI *M (SD)*	37.49 (8.34)
Dietary motivation *M (SD)*	4.30 (0.88)
Social support from family for diet *M (SD)*	3.15 (0.96)
Access to more-healthful foods in the home *M (SD)*	2.90 (0.70)
Access to less-healthful foods in the home *M (SD)*	3.10 (0.67)
Daily servings of fruits *M (SD)*	2.02 (1.05)
Daily servings of vegetables *M (SD)*	1.97 (1.04)
Daily servings of hyperpalatable energy-dense foods *M (SD)*	1.94 (1.43)
Daily servings of sugar-sweetened beverages *M (SD)*	2.01 (2.25)

Six participants were between the 70th and 85th percentile for BMI and were included in the analyses, as this BMI range is associated with increased risk for adolescents transitioning to overweight and obesity (Kimm et al., 2002; Ng & Cunningham, 2020)

### Correlational analyses

Bivariate correlational analyses were performed and presented in Table 2. Results of the correlational analyses revealed several correlations between covariate, predictor, and outcome variables. Adolescent age was negatively associated with social support from family for diet (*r* = − .14, *p* = .026). Adolescent zBMI was positively associated with social support from family for diet (*r* = .19, *p* = .003). Dietary motivation was positively associated with social support for diet (*r* = .49, *p* < .001), access to more-healthful foods in the home (*r* = .24, *p* < .001), daily servings of fruits (*r* = .17, *p* = .008), and daily servings of vegetables (*r* = .14, *p* = .031). Social support for diet was positively associated with access to more-healthful foods in the home (*r* = .26, *p* < .001), access to less-healthful foods in the home (*r* = .14, *p* = .029), and daily servings of vegetables (*r* = .13, *p* = .049). Access to more-healthful foods in the home was positively associated with less access to less-healthful foods in the home (*r* = .47, *p* < .001), daily servings of fruits (*r* = .27, *p* < .001), vegetables (*r* = .24, *p* < .001), energy-dense food (*r* = .14, *p* = .033), and sugar-sweetened beverages (*r* = .23, *p* < .001). Access to less-healthful foods in the home were positively associated with daily servings of fruits (*r* = .30, *p* < .001), vegetables (*r* = .19, *p* = .004), energy-dense food (*r* = .50, *p* < .001), and sugar-sweetened beverages (*r* = .48, *p* < .001). Daily servings of fruits were positively associated with daily servings of vegetables (*r* = .44, *p* < .001) and sugar-sweetened beverages (*r* = .22, *p* < .001). Daily servings of vegetables were positively associated with daily servings of energy-dense foods (*r* = .14, *p* = .026) and sugar-sweetened beverages (*r* = .21, *p* = .001). Daily servings of energy-dense foods were positively associated with daily servings of sugar-sweetened beverages (*r* = .48, *p* < .001).

**Table 2:** Bivariate correlations of study variables

	1	2	3	4	5	6	7	8	9	10
Adolescent age	–									
Adolescent zBMI	− .116	–								
Dietary motivation	− .116	.001	–							
Social support for diet	− .143*	.193**	.487***	–						
Access to more-healthful foods	.006	− .024	.244***	.256***	–					
Access to less-healthful foods	− .090	− .061	.065	.141*	.466***	–				
Daily servings of fruits	− .055	.006	.172**	.122	.269***	.299***	–			
Daily servings of vegetables	− .084	− .065	.140*	.128*	.237***	.186**	.439***	–		
Daily servings of energy-dense foods	− .070	− .064	− .113	.001	.138*	.500***	.126	.144*	–	
Daily servings of sugar-sweetened beverages	− .057	.082	.019	.056	.230***	.480***	.220***	.209**	.483***	–

*p < .05, **p < .01, ***p < .001

### Daily fruit consumption

Table 3 presents the multiple linear regression for ecological predictors of daily fruit consumption. The overall model was significant, *F* (9,224) = 3.74, *p* < .001, *R*^*2*^ = 0.10. Access to less-healthful foods in the home was positively associated with daily fruit consumption (*B* = 0.24, *SE* = 0.08, *p* = .002). As access to less-healthful foods in the home increases by 1-unit, daily fruit consumption increases by an average of 0.24 servings. There were no other significant predictors of daily fruit consumption in the model.

**Table 3:** Ecological predictors of daily fruit consumption

	Estimate	SE	*t*	*p*
Intercept	1.904	0.297	6.417	< .001
Adolescent age	− 0.014	0.038	− 0.377	.706
Adolescent sex	− 0.057	0.138	− 0.411	.682
Adolescent zBMI	0.060	0.138	0.437	.663
Parent marital status	0.012	0.147	0.080	.936
Parent income	0.024	0.147	0.165	.869
Dietary motivation	0.143	0.078	1.853	.065
Social support for diet	− 0.021	0.080	− 0.259	.796
Access to more-healthful foods	0.148	0.080	1.870	.063
Access to less-healthful foods	0.240	0.078	3.100	.002**

*p < .05, **p < .01, ***p < .001

### Daily vegetable consumption

Table 4 presents the multiple linear regression for ecological predictors of daily vegetable consumption. The overall model was significant, *F* (9,224) = 2.43, *p* = .012, *R*^*2*^ = 0.05. Access to more-healthful foods in the home was positively associated with daily vegetable consumption (*B* = 0.18, *SE* = 0.08, *p* = .026). As access to more-healthful foods in the home increases by 1-unit, daily vegetable consumption increases by an average of 0.18 servings. There were no other significant predictors of daily vegetable consumption in the model.

**Table 4:** Ecological predictors of daily vegetable consumption

	Estimate	SE	t	*p*
Intercept	2.433	0.296	8.219	< .001
Adolescent age	− 0.046	0.038	− 1.212	.227
Adolescent sex	− 0.183	0.138	− 1.327	.186
Adolescent zBMI	− 0.159	0.138	− 1.150	.251
Parent marital status	− 0.058	0.146	− 0.392	.695
Parent income	− 0.071	0.147	− 0.485	.628
Dietary motivation	0.025	0.078	0.329	.743
Social support for diet	0.091	0.080	1.142	.255
Access to more-healthful foods	0.177	0.079	2.237	.026*
Access to less-healthful foods	0.061	0.077	0.788	.432

*p < .05, **p < .01, ***p < .001

### Daily hyperpalatable, energy-dense food consumption

Table 5 presents the multiple linear regression for ecological predictors of daily hyperpalatable energy-dense food consumption. The overall model was significant, *F* (9,224) = 9.56, *p* < .001, *R*^*2*^ *=* 0.25. Dietary motivation was negatively associated with daily energy-dense food intake (*B* = -0.24, *SE* = 0.10, *p* = .011). As dietary motivation increases by 1-unit, daily energy-dense food consumption decreases by an average of 0.24 servings. Access to less-healthful foods was positively associated with daily energy-dense food intake (*B* = 0.75, *SE* = 0.10, *p* < .001). As access to less-healthful foods increases by 1-unit, daily energy-dense food consumption increases by an average of 0.75 servings. There were no other significant predictors of daily energy-dense foods consumption in the model.

**Table 5:** Ecological predictors of daily hyperpalatable energy-dense (high fat, high sugar) food consumption

	Estimate	SE	t	*p*
Intercept	2.302	0.363	6.335	< .001
Adolescent age	− 0.033	0.047	− 0.711	.478
Adolescent sex	− 0.279	0.169	− 1.648	.101
Adolescent zBMI	− 0.113	0.169	− 0.669	.505
Parent marital status	− 0.145	0.180	− 0.809	.419
Parent income	0.092	0.180	0.513	.609
Dietary motivation	− 0.243	0.095	− 2.551	.011*
Social support for diet	0.074	0.098	0.758	.450
Access to more-healthful foods	− 0.113	0.097	− 1.163	.246
Access to less-healthful foods	0.746	0.095	7.851	< .001***

*p < .05, **p < .01, ***p < .001

### Daily sugar-sweetened beverage consumption

Table 6 presents the multiple linear regression for ecological predictors of daily sugar-sweetened beverage consumption. The overall model was significant, *F* (9,224) = 8.61, *p* < .001, *R*^*2*^ *=* 0.23. Adolescent zBMI was positively associated with daily sugar-sweetened beverage intake (*B* = 0.84, *SE* = 0.40, *p* = .038). As adolescent zBMI increases by 1-unit, daily sugar-sweetened beverage consumption increases by an average of 0.84 servings. Access to less-healthful foods was positively associated with daily sugar-sweetened beverage intake (*B* = 1.48, *SE* = 0.23, *p* < .001). As access to less-healthful foods increases by 1-unit, daily sugar sweetened beverage consumption increases by an average of 1.48 servings. There were no other significant predictors of daily sugar-sweetened beverage consumption in the model.

**Table 6:** Ecological predictors of daily sugar-sweetened beverage consumption

	Estimate	SE	t	*p*
Intercept	2.356	0.861	2.737	.007
Adolescent age	-0.000	0.111	0.000	1.0
Adolescent sex	0.446	0.401	1.111	.186
Adolescent BMI	0.838	0.400	2.093	.038*
Parent marital status	-0.004	0.427	-0.011	.991
Parent income	-0.812	0.423	-1.903	.058
Dietary motivation	0.033	0.225	0.145	.885
Social support for diet	-0.136	0.233	-0.583	.561
Access to more-healthful foods	0.175	0.230	0.762	.447
Access to less-healthful foods	1.478	0.225	6.567	< .001***

Results did not significantly change when performing these secondary data analyses with the six participants who fell below the 85th percentile for BMI excluded from the models; however, zBMI no longer significantly predicted daily sugar-sweetened beverage intake (*B* = 0.84, *SE* = 0.43, *p* = .050)

*p < .05, **p < .01, ***p < .001

## Discussion

The purpose of this study was to examine the ecological determinants of daily dietary intake patterns in Black adolescents with overweight and obesity. It was hypothesized that higher levels of dietary motivation, higher levels of social support for healthful eating, greater access to more-healthful foods and lower access to less-healthful foods in the home are associated with more self-reported daily intake of fruits and vegetables and lower self-reported daily consumption of hyperpalatable energy-dense food (high fat, high sugar) and sugar-sweetened beverages. The results demonstrated that greater access to more-healthful foods was associated with increased daily vegetable intake. We also found that greater access to less-healthful foods was associated with increased daily energy-dense food intake, whereas increased self-reported dietary motivation was associated with decreased daily energy-dense food intake. Additionally, greater access to less-healthful foods was associated with increased sugar-sweetened beverage consumption. These findings suggest that access to more-healthful foods is associated with more healthful dietary intake, while lower access to less-healthful foods and increased motivation for more healthful eating were associated with less unhealthful eating among Black adolescents. Taken together, findings from the current study are largely consistent with previous literature on dietary outcomes; however these findings are novel and extend previous research as this was the first study to examine adolescent self-reported daily fruit and vegetable, energy-dense foods, and sugar-sweetened beverage intake using an ecological framework that examines the combined influence of adolescent dietary motivation, social support for diet from family, and access and availability of healthful foods in the home in Black adolescents with overweight and obesity. Importantly, these findings revealed ecological influences of daily dietary habits among Black adolescents with overweight and obesity with high BMI, and further advances understanding the determinants of obesity among Black youth with increased vulnerabilities to health outcomes related to poor dietary outcomes.

In this study, greater access to more-healthful foods in the home predicted greater daily consumption of vegetables. This finding is largely consistent with prior studies, as most studies have documented associations between greater access to healthful foods and greater vegetable consumption (Cho & Kim, 2018; Granner, 2011; Loth et al., 2016). The finding that greater access to less-healthful foods predicted more daily intake of fruit is surprising and was opposite of our hypothesis. Prior research has also demonstrated associations between greater access to more-healthful foods in the home and greater fruit consumption (Amuta et al., 2015; Granner, 2011; Loth et al., 2016). Although access to more-healthful foods was not significant, there was a strong and significant association between access to less-healthful foods and access to more-healthful foods in this study. It may be likely that higher levels of food access in general may predict greater consumption of fruit. Other factors may also explain this discrepant finding, which include individual and cultural preferences for consuming fruit, such as taste preferences and traditional and cultural practices, as well as acceptability and knowledge on preparing and serving fruits at meals, however, much research is warranted to understand this finding in light of access and availability of foods in the home (Di Noia & Byrd-Bredbenner, 2013; Grigsby-Toussaint et al., 2010; Winham et al., 2020).

Interestingly, this study found that higher levels of dietary motivation were associated with lower consumption of energy-dense foods whereas higher levels of access to less-healthful foods in the home were associated with greater consumption of energy-dense foods. Unlike our findings with fruit and vegetable consumption, it appears that limited access to less-healthful foods in the home and adolescent dietary motivation are important predictors of adolescent energy-dense food intake when considering multiple ecological factors in the model. Previous research has demonstrated that greater access to more-healthful foods in the home and higher adolescent dietary motivation is associated with higher diet quality in adolescents (Cho & Kim, 2018; Granner, 2011; Parks et al., 2018; Zhang et al., 2020). One potential explanation is that it may be more feasible for adolescents to be motivated to eat less energy-dense food if there are less unhealthful foods in the home, as it may be more challenging for adolescents to develop dietary motivation if there is restricted access to fruits and vegetables in the home.

Across all outcomes, social support was not a predictor of daily dietary intake, which is somewhat inconsistent with previous literature. Previous research from our group has demonstrated positive associations between social support for diet and quality of family meals over 16-weeks (Quattlebaum et al., 2025). Findings from the larger FIT trial demonstrated that higher levels of parental responsiveness and lower levels of parental demandingness were associated with higher frequency of weekly family mealtimes over 16 weeks in families participating in the group-based motivational plus family weight loss intervention (Wilson et al., 2021). One potential explanation for why social support was not a predictor of daily dietary intake in this study is that other factors, such as access and motivation, may have a greater influence on dietary outcomes related to fruit and vegetable and foods that are high in fat and sugar. Black families often report preparing more pre-prepared meal and serving more fast foods at meals and serving less fruits and vegetables at meals and report having fewer access to more healthful foods in the home (Berge et al. 2016a). With limited access and availability of healthful foods in the home, it may be difficult for adolescents to feel supported towards eating a more healthful diet if there are no opportunities to engage in these behaviors. Similarly, as adolescent develop greater dietary autonomy, they may require less support from their parents around engaging in dietary behaviors. While previous studies have demonstrated that greater parent social support, including encouragement, are associated with increased dietary quality in youth, some studies have also suggested that higher levels of authoritarian parenting are more predictive of healthier eating and adherence to dietary guidelines in Black adolescents (Ardakani et al., 2024). More nuanced approaches to understanding social support for diet that both tangible supports, such as assistance in preparing healthful meal, and encouragement for maintaining heathier diets, are warranted (Berge et al. 2016b; Larson et al. 2015; Watts et al. 2017).

Findings from this study also revealed that greater access to less-healthful foods in the home and higher BMI was associated with increased daily intake of sugar-sweetened beverages. These findings suggest that among African American adolescents with the highest BMIs (95%) and above, had higher positive associations with sugar-sweetened beverages intake. Prior research has indicated that the greater consumption of sugar-sweetened beverages is associated with increased obesity risk among youth (Nguyen et al., 2023). Black adolescents with overweight and obesity with increased access to sugar-sweetened beverages and other high-caloric, nutrient deficient foods may be at a greater risk for greater BMI, and thus, a greater risk for chronic disease related to obesity and poor nutrition (Lane et al., 2024). Future research is needed to understand the various contributing factors associated with increased access to sugar-sweetened beverages in the home. Understanding the association of sugar-sweetened beverage intake among obesity risk in Black youth, may include assessing adolescent and parent perceptions about the healthfulness of sugar-sweetened beverages, cost of sugar-sweetened beverages, and biased marketing and advertisements (Munsell et al., 2016; Okoli et al., 2024; Roesler et al., 2021).

This study has limitations. This study was cross-sectional are as a result, only associations can be made between variables of interests. More longitudinal studies are needed to determine causality, and it may be possible that changes in ecological predictors in the study influence dietary outcomes over time, for instance, Quattlebaum and colleagues (2025) demonstrated that the higher levels of parent social support for diet over time predicted higher atmosphere quality at family mealtimes in Black youth (Quattlebaum et al., 2025). Another limitation is that other ecological factors may be important predictors of adolescent dietary outcomes that warrant further examination, for instance, Di Noia and colleagues (2013) demonstrated that the combination of taste preferences for fruit and vegetables and greater access to fruit and vegetables in the home predicted greater intake of these foods in African American adolescents (Di Noia & Byrd-Bredbenner, 2013). Future studies should also consider other contextual influences of adolescent diet, for instance, adolescents may also receive other support for diet outside of the home, such as from peers and school and that they may be engaging in more eating occasions that provide differential opportunities for engaging in more healthful or less healthful eating outside of the home (Story et al., 2002).

Despite these limitations, there are several strengths of the study. This study examined dietary outcomes across a range of food types, which helps provide a more contextual understanding of dietary outcomes across different food groups in Black adolescents with overweight and obesity. In addition, this study assessed key ecological factors that have not been examined together but which are highly supported by theory and have been shown to individually predict diet quality in general among adolescents. These findings from this study suggest that access and dietary motivation are more salient predictors of dietary outcomes in Black adolescents with overweight and obesity when considering the combined influence of multiple ecological factors. Measures from this study were all adolescent-self report, which provides greater insight into understanding adolescent dietary behaviors and ecological influence beyond parent-reported measures. Finally, this study exclusively sampled Black adolescents with overweight and obesity, which despite the limited generalizability to other samples, extends previous literature by examining this underserved and high-risk group. In summary, these findings may inform future health promotion programs across various ecological levels of dietary behaviors in Black youth with overweight and obesity.

## Data Availability

The dataset used for the current study is available from the corresponding author on reasonable request.

## Declarations

### Trial registration

The FIT trial was registered on January 23, 2013, on ClinicalTrials.gov NCT01796067.
